# Evaluation of CADD Scores in Curated Mismatch Repair Gene Variants Yields a Model for Clinical Validation and Prioritization

**DOI:** 10.1002/humu.22798

**Published:** 2015-05-20

**Authors:** K. Joeri van der Velde, Joël Kuiper, Bryony A. Thompson, John‐Paul Plazzer, Gert van Valkenhoef, Mark de Haan, Jan D.H. Jongbloed, Cisca Wijmenga, Tom J. de Koning, Kristin M. Abbott, Richard Sinke, Amanda B. Spurdle, Finlay Macrae, Maurizio Genuardi, Rolf H. Sijmons, Morris A. Swertz, InSiGHT Group

**Affiliations:** ^1^ Genomics Coordination CenterUniversity Medical Center GroningenUniversity of GroningenGroningenThe Netherlands; ^2^ Department of GeneticsUniversity Medical Center GroningenUniversity of GroningenGroningenThe Netherlands; ^3^ Department of EpidemiologyUniversity Medical Center GroningenUniversity of GroningenGroningenThe Netherlands; ^4^ Department of Genetics and Computational BiologyQIMR Berghofer Medical Research InstituteBrisbaneAustralia; ^5^ Department of Colorectal Medicine and GeneticsRoyal Melbourne HospitalMelbourneAustralia; ^6^ Department of MedicineThe Royal Melbourne HospitalUniversity of MelbourneMelbourneAustralia; ^7^ Institute of Medical Genetics“A. Gemelli” School of MedicineCatholic University of the Sacred HeartRomeItaly

**Keywords:** Lynch syndrome, variant classification, pathogenicity prediction, cumulative link model

## Abstract

Next‐generation sequencing in clinical diagnostics is providing valuable genomic variant data, which can be used to support healthcare decisions. In silico tools to predict pathogenicity are crucial to assess such variants and we have evaluated a new tool, Combined Annotation Dependent Depletion (CADD), and its classification of gene variants in Lynch syndrome by using a set of 2,210 DNA mismatch repair gene variants. These had already been classified by experts from InSiGHT's Variant Interpretation Committee. Overall, we found CADD scores do predict pathogenicity (Spearman's *ρ* = 0.595, *P* < 0.001). However, we discovered 31 major discrepancies between the InSiGHT classification and the CADD scores; these were explained in favor of the expert classification using population allele frequencies, cosegregation analyses, disease association studies, or a second‐tier test. Of 751 variants that could not be clinically classified by InSiGHT, CADD indicated that 47 variants were worth further study to confirm their putative pathogenicity. We demonstrate CADD is valuable in prioritizing variants in clinically relevant genes for further assessment by expert classification teams.

## Introduction

Reliable estimation of gene variant pathogenicity, especially for missense variants and small in‐frame insertions/deletions (indels), is a major challenge in clinical genetics. This challenge is now being exacerbated by the introduction of next‐generation sequencing in clinical diagnostics, which is identifying large numbers of candidate disease‐causative variants, ranging from about 250 [Lohmann and Klein, 2014], to 400–700 [Yang et al., 2013], up to a mean of 1,083 [Saunders et al., 2012] variants per exome, depending on which filter steps and stringency are applied. Since it is not feasible to perform functional analysis of each variant, in silico tools have become an important tool in assessing variant pathogenicity. Unfortunately, although there are many potential methodologies and tools [Cooper and Shendure, 2011], they often lack clinical validation. As the adaptation of high‐throughput sequencing in clinical practice increases, the need for standardized, validated, and easy‐to‐use in silico classification tools is becoming even more pressing [Saunders et al., 2012; Yang et al., 2013].

The recently launched Combined Annotation Dependent Depletion (CADD) [Kircher et al., 2014] method offers a standardized, genome‐wide, variant scoring metric (C‐score) that incorporates the weighted results of widely used in silico pathogenicity prediction tools, such as SIFT [Kumar et al., 2009] and PolyPhen [Adzhubei et al., 2010], and of genomic annotation sources like ENCODE [Dunham et al., 2012]. The resulting CADD scores are expressed as a measure of deleteriousness (selection pressure bias) for single‐nucleotide variants (SNVs) and small indels. A high score represents variants that are not stabilized by selection, which are more often disease‐causing than expected by random chance [Kircher et al., 2014]. In contrast, a low score indicates that a variant resembles evolutionary stable, commonly occurring genetic variation that poses no apparent disadvantage for an organism. The scores were shown to correlate strongly to known variant pathogenicity, such as those causing a predisposition to autism spectrum disorders, intellectual disability, thalassemia, and more broadly to pathogenic variants taken from the NHGRI GWAS catalog [Welter et al., 2013] and ClinVar [Landrum et al., 2013] database. To make interpretation and comparison easier, C scores are logarithmically ranked to form scaled C‐scores, similar to how PHRED scores are used in the FASTQ format.

As an easy‐to‐use resource that brings out the predictive power of many programs and data combined, CADD may replace the plethora of tools currently being used. However, before considering implementation of CADD in clinical work, it is important to evaluate and validate its utility by comparing its outcome with that of existing, consistent, large‐scale expert assessments.

The Variant Interpretation Committee (VIC) is an expert panel of the International Society for Gastrointestinal Hereditary Tumours (InSiGHT). They conducted a thorough clinical classification of 2,360 variants (as of February 2014) in the DNA mismatch repair (MMR) genes *MLH1* (MIM #120436), *MSH2* (MIM #609309), *MSH6* (MIM #600678), and *PMS2* (MIM #600259) that had been identified in patients suspected of having Lynch syndrome [Thompson et al., 2013b]. This cancer predisposition syndrome, previously known as hereditary nonpolyposis colorectal cancer, is caused by DNA MMR deficiency.

The InSiGHT variant classification method is based on a combination of clinical and experimental (molecular) evidence, such as family history and cosegregation with the disease, tumor findings, population allele frequencies, and mRNA/protein functional assays (in accordance with established guidelines, available at http://www.insight‐group.org/criteria).

The variants were classified following a five‐tier system [Plon et al., 2008], with class descriptions as follows:

- Class 1: not pathogenic/no clinical significance.
- Class 2: likely not pathogenic/little clinical significance.
- Class 3: uncertain clinical significance.
- Class 4: likely pathogenic.
- Class 5: pathogenic.

Variants that cannot be placed in classes 1, 2, 4, or 5 based on existing evidence are assigned to class 3 by default and are considered variants of uncertain clinical significance. It is recognized [Thompson et al., 2013b] that class 3 may include some cases with conflicting evidence.

Here, we investigate whether CADD scores are concordant with variant classifications assigned by the InSiGHT VIC. We show that, overall, CADD and InSiGHT yield similar results, but that there are also some important discordant cases. Our contributions in this paper are:

1. An extensive evaluation of agreement between the in silico CADD predictions and the InSiGHT expert classifications of variant pathogenicity.
2. Detection and assessment of conflicting classifications.
3. A CADD‐based prioritization of variants of uncertain clinical significance.
4. Assessment of the reliability of CADD for use in a clinical setting.

These contributions shed light on an important question in clinical genetic diagnostics: are bioinformatics tools powerful enough to enable genome‐wide variant interpretation without loss of quality when compared with classification by clinical expert panels that can also take into account a range of clinical and molecular data relevant for specific genetic diseases?

## Materials and Methods

### Data Processing

We downloaded 2,744 variants (as of February 2014) from the InSiGHT LOVD database (at http://chromium.liacs.nl/LOVD2/colon_cancer/) for *MLH1*, *MSH2*, *MSH6*, and *PMS2*. RefSeq identifiers NM_000249.3, NM_000251.2, NM_000179.2, and NM_000535.5 were added to the cDNA position. This allowed the successful conversion of 2,582 variants to genomic DNA notation in VCF format by running Ensembl VEP5 [McLaren et al., 2010]. CADD (version 1.0) was able to score 2,580 of those (NM_000249.3:c.1254T>R and NM_000535.5:c.1875A>Y failed). Of these 2,580 variants, 370 were not assessed by InSiGHT, or in a few cases belonged to multiple classes. This means that 2,210 variants were classified and belong to one of the five classes of the International Agency for Research on Cancer (IARC) five‐tiered classification system: 151 variants belong to class 1 (not pathogenic), 84 to class 2, 751 to class 3, 181 to class 4, and 1,043 to class 5 (pathogenic).

In addition, we ran SnpEff to obtain functional effect predictions using canonical transcript references and an upstream downstream interval length of five bases. The output was curated to reduce the number of effects from two to one in the case of both INTRON and SPLICE_SITE “effects,” by removing the INTRON effect. We used NM_000251.2 for MSH2 (whereas the LOVD was based on NM_000251.1) to enable ENSEMBL VEP to process the data, without issues (out of 920 MSH2 variants, 855 were successfully converted to VCF/gDNA notation).

### Cumulative Link Model

To detect discrepancies between the CADD scores and the InSiGHT classification, we assumed that a partitioning of the scores would exist. In other words, the continuous C‐scaled scores can be binned into the ordinal IARC classes. Working on this assumption, we were able to define a cumulative link model (ordinal regression) [McCullagh, 1980; Agresti, 2002]. In a cumulative link model, an ordinal response variable *Y_i_* can fall in j=1,...,J ordered classes. This response variable *Y_i_* then follows a distribution with parameter *π_i_* where *π_ij_* denotes the probability that the *i*th observation falls in the *j*th response class (such that $\sum_{j\;=\;1}^{J}\pi_{ij}=1$). Since we are dealing with individual observations (instead of counts), the categorical distribution is used, which can be viewed as a special case of the multinomial distribution of *n* observations $Y_{i}∼\mathrm{Mult}\left(n,\pi_{i}\right)$ with n = 1:

Yi∼ Categorical πi

The cumulative probability is then defined as:

$$\gamma_{ij}=P\left(Y_{i}\leq j\right)=\pi_{i1}+...+\pi_{ij}$$

Here, we considered a proportional odds model, using a logit link function:  logit (p)= log [p/(1−p)]. The cumulative logits for all but the last class, j=1,...,J−1, are then defined as:

logit γij= logit PYi≤j= log PYi≤j1−PYi≤j

This gives a regression for the cumulative logits:

logit γij=θj−xi⊤β

where *θ_j_* represents the logit‐scaled cut‐off for class *j*, *x_i_* being the vector of explanatory variables for the *i*th observation, and *β* is the corresponding set of regression parameters. Note that $x_{i}^{⊤}\beta$ does not contain an intercept. The parameters *θ_j_* act as a set of continuous “cut‐off points”, such that $-\infty <\theta_{1}<\cdots <\theta_{J-1}<\infty$. To assess the probability that the *i*th observation falls within one of ordinal response classes *j*, we can write:

$$P\left(Y_{i}=j|x_{i}^{⊤}\beta \right)=\left\{\begin{array}{cc} \gamma_{ij}, & j=1\\ \gamma_{ij}-\gamma_{i\left(j-1\right)}, & j=2,...,\\ 1-\gamma_{i\left(J-1\right)}, & j=J \end{array}\;\;\;\;\;\;\right.J-1$$

**Figure 1:**
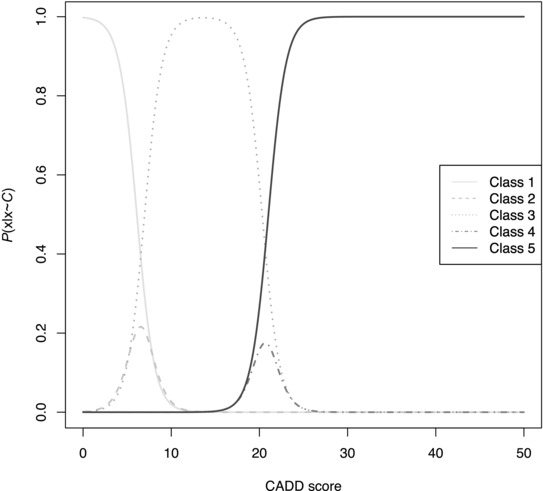
Probability that a CADD score will belong to a certain InSiGHT class. The inverse logit (logit^−1^) was applied to each of the response variables. Classes 2 and 4 are dominated by class 3 under this model.

We used the CADD score as an explanatory variable for the ordinal response of InSiGHT. The parameters were estimated using JAGS, a program for analysis of Bayesian graphical models using Gibbs sampling [Plummer, 2003]. Convergence of the Markov Chain Monte Carlo inference was assessed using the potential scale reduction factor [Gelman and Rubin, 1992; Plummer et al., 2006]. Figure 1 shows the probability that a given CADD score belongs to a certain InSiGHT class by using the posterior distributions for *θ* after convergence. Discrepancies were detected by analyzing the deviance of the observations. Deviance can be thought of as a measure of “surprise”, how likely a certain observation is under the fitted parameters of the model. Formally:

DYi,θ^=−2 log [P(Yi|θ^)]

with *Y_i_* being the observation and $\hat{\theta }$ the parameters of the fitted model. Observations of *θ* corresponding to variants in the 95th percentile of the mean deviance—those with the highest deviance—were re‐examined.

### Data Availability

The data and scripts used in this paper can be downloaded from: http://molgenis.org/downloads/vdVelde_Kuiper_etal_2015/

## Results

### Exploratory Data Analysis

**Figure 2:**
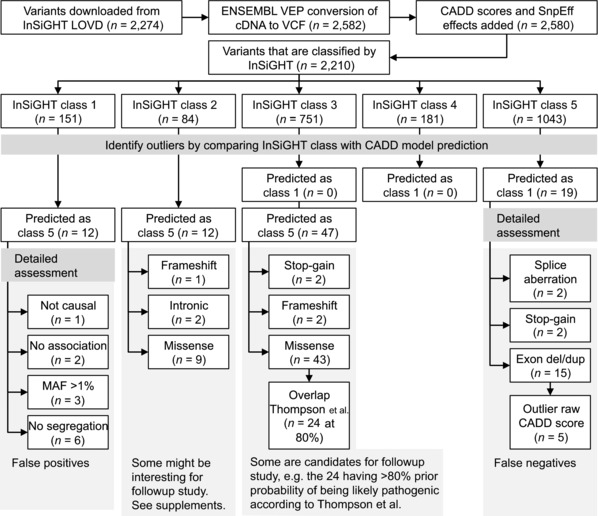
Flowchart describing the steps and results of the analysis.

We calculated the CADD scores for 2,744 MMR gene variants that were downloaded from the InSiGHT group LOVD (available at http://chromium.liacs.nl/LOVD2/colon_cancer). A total of 534 variants had to be omitted, either because converting the complementary DNA HGVS nomenclature [den Dunnen and Paalman, 2003] based notation to genomic DNA VCF (Variant Call Format version 4.0 [Danecek, 2011]) based notation failed (162 variants), or the CADD scores could not be unambiguously assigned (two variants with *T*>*R* and *A*>*Y* substitutions), or because they had not yet been classified by the InSiGHT VIC (i.e., they were recent submissions, or not reported as germline variants [370 variants]). See Figure 2 and *Methods and Materials* for details. The 2,210 remaining variants fell within one of the five classes: class 1 (*n* = 151), class 2 (*n* = 84), class 3 (*n* = 751), class 4 (*n* = 181), or class 5 (*n* = 1,043).

**Figure 3:**
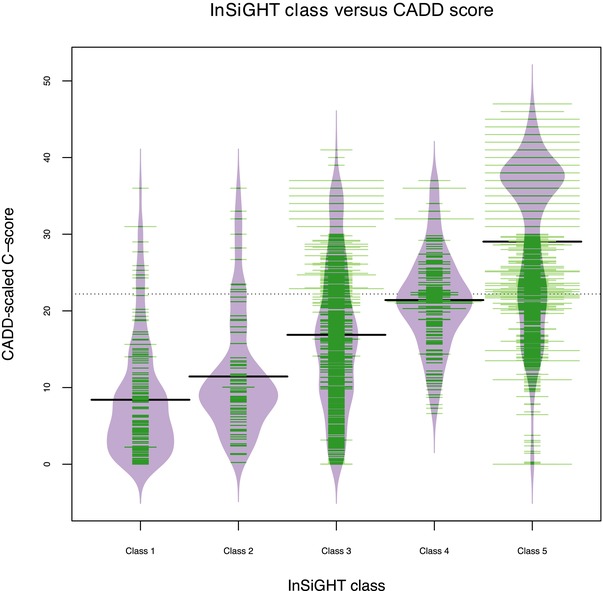
Beanplot [Kampstra, 2008] showing the data points (green) and density estimation (purple) of the scaled CADD C‐score per InSiGHT class. The width of the green lines is relative to the number of data points at that score. Black horizontal lines indicate the mean per InSiGHT class; the dotted line shows the overall mean. The mean scores of classes 1–5 show a respective stepwise increase of 8.41 (*σ* = 7.46), 11.44 (*σ* = 7.72), 16.87 (*σ* = 9.40), 21.41 (*σ* = 6.13), and 29.04 (*σ* = 10.28). The unclassified group (class 3) shows a flatter distribution than the other classes.

Overall, the CADD‐scaled C‐score distributions for each class correlate with the InSiGHT classification (Spearman's *ρ* = 0.595, *P* < 0.001). In Figure 3, the distribution of the scores per class is represented in a beanplot [Kampstra, 2008]. See also Supp. Figures S1–S4 for CADD scores of the InSiGHT variants for each gene, using known variants identified in the Genome of the Netherlands [TGotN Consortium, 2014a, 2014b] and 1000 Genomes [T1GP Consortium, 2012] projects as population background reference.

### Discrepancy Assessment

Using a Bayesian cumulative link model, we identified 108 (4.89% of 2,210) cases for which a different class would be assigned (see *Materials and Methods*). Further analysis focused on the cases for which the nonpathogenic (class 1) and pathogenic (class 5) classifications were reversed, as these suggested major disagreements between CADD and the InSiGHT VIC verdict (see Table 1). The explanations per variant for this analysis can be found in Supp. Table S1.

**Table 1:** Number of InSiGHT Variants Reassigned to Alternative Classes According to the Cumulative Link Model Fitted on CADD Scores

	InSiGHT classification				
CADD model	Class 1	Class 2	Class 3	Class 4	Class 5
Class 1	135				19
Class 2		71			
Class 3	4	1	704	3	3
Class 4				171	
Class 5	12	12	47	7	1021

### False Positives

We identified 12 variants (0.54% of 2,210) that were classified as nonpathogenic (class 1) by the InSiGHT VIC, but they were predicted to be pathogenic (class 5) according to the CADD‐based cumulative link model (see *Materials and Methods*). Re‐examination of the available data for these variants strongly supports the original InSiGHT classification based on the following evidence:

- Segregation data are inconsistent with the variant being a dominant, high‐risk, pathogenic sequence variant in pedigrees (likelihood ratio ≤0.01).
- Variant with reported frequency ≥1% in the general population (1000 Genomes Project), and no evidence that variant is a founder mutation.
- These are not high‐risk variants that are uniquely associated with Lynch syndrome (they have also been seen in individuals who do not meet the international criteria for Lynch syndrome).
- Variant leads to a known attenuated protein function, but this does not cause Lynch syndrome (it has also been seen in healthy individuals and there is a lack of evidence for MMR deficiency as shown by MSI and immunohistochemical testing).

Although these explanations are specific to Lynch syndrome‐related variants, they indicate that CADD might overestimate the general pathogenicity of some variants. Most overestimations could be easily resolved in a clinical Standard Operating Procedure (SOP) by using population allele frequency as a filter or incorporating the use of patient pedigree analysis data; these are already common practices in many clinical laboratories. The remainder could be resolved by incorporating more in‐depth findings from validated protein functional assays or from risk estimates based on large, well‐designed, case‐control studies that consider cohort size, geography/ethnicity, and quality control measures [Thompson et al., 2013b]. An evaluation of likely not pathogenic (class 2) variants predicted to be pathogenic (class 5) can be found in Supp. Table S2.

### False Negatives

We identified 19 cases (0.86% of 2,210) for which the cumulative link model predicted the respective variants to be class 1, whereas InSiGHT scored them as class 5. This indicates that the model might also underestimate effects. Similar to the approach to the false positives, outlined above, our re‐examination of these variants supported the original InSiGHT classification.

CADD scores are developed for scoring any possible human SNVs or small indels [Kircher et al., 2014]. It was therefore expected that large structural variants would be missed or inaccurately scored (for 5/15 structural variants) by CADD. To simplify the interpretation, the scaled C‐scores are based on the rank of the C‐score relative to all the C‐scores for 8.6 billion possible SNVs. Typical variant C‐scores in this study ranged from −4 to 14, whereas the five structural variants in question scored very highly (between 350 and 550), which was expected considering the likely pathogenicity of exon deletions relative to missense variants or codon deletions, for example. However, the scaling algorithm seems to fail for such extreme C‐scores, and this results in reverting the score for the respective variant into a very low‐scaled C‐score instead. We applied SnpEff [Cingolani et al., 2012] as a second‐tier test. This tool has been developed to annotate and predict the effects of variants in genes in a robust and qualitative way, thereby complementing the quantitative nature of CADD scores. Using SnpEff, we were able to correct 17 of the 19 false‐negative cases. SnpEff recognized 14 of the 15 structural variants, most as “EXON_DELETED,” one of two splice aberrations as “FRAME_SHIFT,” and two of two truncating mutations as “STOP_GAINED.” These effect types are annotated as HIGH impact in SnpEff, in contrast to MODIFIER, LOW, or MODERATE effect types. By using SnpEff information, we have shown that CADD results should be complemented by this tool, or a comparable tool, to compensate for sporadic underestimations. See Supp. Figure S5 for an overview of SnpEff variant effect predictions in relation to CADD scores and InSiGHT classifications.

### Variants of Unknown Significance

Class 3 mainly contains variants for which insufficient clinical or molecular data are available, but also a limited number of variants that have discordant findings (i.e., are resistant to classification). Most of these variants can easily be assigned to another class as soon as more data become available. As expected, the distribution of the CADD scores for class 3 variants, as visualized in Figure 3, is much flatter than the distributions for the other classes. Matching the CADD score of each class 3 variant to the distributions of the other classes (and thus, the likelihood of belonging to one of them) allows us to propose an endpoint classification that is, according to the model, more likely than belonging to class 3 for these variants. In other words, we can suggest prioritization of a variant for reclassification (using additionally obtained clinical and molecular evidence) when its CADD score deviates far enough from this mean, reaching a score that falls into the distributions of known nonpathogenic or pathogenic variant classes (see *Materials and Methods*).

We performed this analysis and 47 variants (2.13% of 2,210) that the InSiGHT VIC classified as class 3 (uncertain significance) had CADD scores of ≥34, which fell in the >99% probability range for known class 5 (pathogenic) variants (see Fig. 1). Of these 47 variants, 43 were missense with a mean CADD score of 35.33 (*σ* = 1.04, 27 in *MLH1*, 10 in *MSH2*, four in *MSH6*, and two in *PMS2*). The remaining four were truncating mutations: two stop‐gain variants (c.2250C>A and c.2250C>G, both with a CADD score of 41), and two frameshift variants (c.2252_2253del and c.2262del) with CADD scores of 39 and 40. These four variants are all located in the *MLH1* gene; they were classified as class 3 by the InSiGHT VIC due to insufficient evidence, because the stop codons are introduced in the last exon (19) and are located outside any known functional domains.

We compared these findings with the previous use of a prediction model [Thompson et al., 2013a] on 481 substitutions [Thompson et al., 2013b] of uncertain effect. In this analysis, 173 InSiGHT missense variants of uncertain significance (class 3), with a >80% probability in favor of pathogenicity, were prioritized for further investigation using multifactorial likelihood analysis. The model calibrated a combination of in silico tools to predict probabilities of pathogenicity, which is conceptually somewhat similar to the way CADD scores are constructed, except here the model was specifically for MMR gene variants associated with Lynch syndrome.

By comparing the two sets of results, that is, the 173 previously identified variants with our 43 prioritized variants, we found an overlap of 24 variants (see Table 2). Since they were called by both models, we consider these 24 missense variants to be the most urgent candidates for further research to determine their pathogenicity. Of the remaining 19 variants prioritized uniquely by CADD, 17 had been evaluated before with prior probabilities of pathogenicity ranging from 7% (MSH2:c.1418C>T) to 74%–76% (MLH1: c.85G>T, c.187G>A, c.299G>A, c.794G>A, c.955G>A, c.1976G>A, PMS2: c.137G>A).

**Table 2:** The 24 Variants That Are Still Uncertain and Predicted by Bioinformatic Tools to Be Likely Pathogenic, According to the Probabilities of the MAPP + PolyPhen2 Calibrated Model [Thompson et al., 2013a] and the CADD Model

Gene	Variant	AA ch.	Previous [Thompson et al., 2013a]	Here
MLH1	c.1037A>G	p.Q346R	0.95	0.99
MLH1	c.109G>A	p.E37K	0.87	0.99
MLH1	c.112A>G	p.N38D	0.94	0.99
MLH1	c.125C>T	p.A42V	0.96	0.99
MLH1	c.184C>A	p.Q62K	0.88	0.99
MLH1	c.1918C>T	p.P640S	0.82	0.99
MLH1	c.1919C>T	p.P640L	0.93	0.99
MLH1	c.304G>A	p.E102K	0.87	0.99
MLH1	c.307G>C	p.A103P	0.97	0.99
MLH1	c.331G>C	p.A111P	0.97	0.99
MLH1	c.347C>A	p.T116K	0.93	0.99
MLH1	c.65G>C	p.G22A	0.89	0.99
MLH1	c.67G>A	p.E23K	0.86	0.99
MLH1	c.74T>C	p.I25T	0.86	0.99
MLH1	c.80G>C	p.R27P	0.97	0.99
MLH1	c.925C>T	p.P309S	0.83	0.99
MSH2	c.1799C>T	p.A600V	0.96	0.99
MSH2	c.1826C>T	p.A609V	0.96	0.99
MSH2	c.2064G>A	p.M688I	0.89	0.99
MSH2	c.2141C>T	p.A714V	0.87	0.99
MSH2	c.2168C>T	p.S723F	0.88	0.99
MSH2	c.2187G>T	p.M729I	0.88	0.99
MSH2	c.529G>A	p.E177K	0.86	0.99
MSH6	c.3682G>C	p.A1228P	0.97	0.99

We also compared a CADD‐based binary classifier for missense variants with the multifactorial likelihood model (see Supp. Text for these results).

## Discussion

We investigated the use of CADD scores for the prediction of clinical classifications by comparing them with a high‐quality clinical data set developed by the InSiGHT VIC, which is based on quantitative and qualitative interpretation of both clinical and molecular data. Generally, the CADD model predictions fitted the InSiGHT classification. Out of the 2210 variants we tested and classified by InSiGHT, we identified 12 (0.54%) nonpathogenic (class 1) variants that the CADD model predicted to be pathogenic (class 5), and 19 variants (0.86%) of class 5 that CADD predicted to be class 1. The difference could be explained by two considerations: the CADD model was not designed to classify large structural or splice‐site variants (55% of all the discordant cases, 89% of the false negatives), and the clinical observations, population allele frequencies, and experimental molecular data sometimes convincingly suggested an alternative interpretation (39% of all discordant cases, 100% of the false positives). CADD's main underestimation of pathogenicity was due to its inability to accurately predict the effects of whole exon deletions or duplications. In five such cases, the C‐score was in fact extremely high, but this was not translated into a high‐scaled C‐score. The use of a second‐tier test, in this case SnpEff, boosted the sensitivity of classifying via CADD by correcting 17 out of 19 of these underestimations.

We showed that estimating the deleteriousness of whole exon deletions/duplications is a weakness of CADD and this needs to be addressed. The InSiGHT data show that such structural variation is often pathogenic, but this is not always recognized by CADD. To avoid incorrect results, and in line with the design limitations of CADD as acknowledged by its authors, we recommend CADD should not be used to judge the pathogenicity of large structural variation as part of an automated variant processing pipeline.

We also investigated the 12 cases of pathogenicity overestimation by CADD, which showed that these false positives could be explained by data used for the InSiGHT classification that was not used for in silico prediction (such as the presence of the variant in the general population or lack of cosegregation of the variant with the disease). These results underscore the importance of using clinical data in the diagnostic interpretation of variants.

There are a few variants in the InSiGHT database with a known negative effect, such as attenuated protein function, that are classified as nonpathogenic. The InSiGHT VIC requires both concordant functional and clinical evidence to assign pathogenicity; they do not accept that attenuated function would necessarily be associated with Lynch Syndrome—or any phenotype for that matter. In our analysis, for example, CADD predicted a deleterious effect for MLH1:c.394G>C, which is indeed known to cause attenuated protein function [Lipkin et al., 2004], but is not considered to be pathogenic in the context of Lynch syndrome because it is not known to be associated with the causal phenotype. Variant classifications such as those currently provided by the InSiGHT VIC for MMR genes are specifically developed for a given phenotype, namely, Lynch syndrome. Therefore, as acknowledged by the VIC [Thompson et al., 2013b], they may not capture modest disease penetrance or other disease phenotypes associated with a given variant. This highlights the fact that some apparent discrepancies may simply be explained by the difference in application of “research tools” such as CADD and “clinical tools” such as the InSiGHT database; the latter focuses on results that are of practical value for a clinical geneticist instead of yielding a spectrum of variants with possible intermediate penetrance that then require further interpretation and individualized risk management protocols.

In general, there is limited added value in using CADD scores to assess truncating variants since they are already known to often be pathogenic for known disease genes. The field of in silico prediction benefits most from the power of CADD scores when they are applied to predict the pathogenicity of nonsynonymous SNVs. Here, we show that CADD performs well on this type of variant for Lynch syndrome, although a disease‐specific model performs better.

We identified 47 variants that had been assigned by InSiGHT to class 3 (uncertain significance), which, according to the CADD model, had a high probability of being pathogenic. Of these, 24 missense variants were already strongly suspected of being pathogenic by a previous in silico study on MMR gene variant classification [Thompson et al., 2013a] and we consider them to be top candidates for further study to confirm their pathogenicity. This suggests that CADD, in a fashion similar to existing disease‐specific pathogenicity prediction models, can help in prioritizing variants for the collection of missing clinical and molecular data.

Taken together, we have shown that CADD scores are in high agreement with expert assessments of MMR gene variant pathogenicity that is based on multiple data sources for quantitative‐multifactorial and qualitative analysis. As expected, CADD scores are not yet suitable to interpret large structural variants such as deletions and duplications of exons. Other underestimation effects are rare and often detectable with a second‐tier test. Any overestimated variants could be excluded based on population frequency, cosegregation analyses, or evidence showing no association or causality.

Calibrated in silico pathogenicity prediction models are not intended to replace functional wet‐laboratory studies, but are instead complementary methods to let clinics benefit from existing gold standard classifications, by accessing their expert knowledge and making it possible to assess and prioritize novel variants with reasonable confidence, without the need for often unfeasible amounts of laboratory work. We believe CADD fits this translatory role very well, particularly because of its generic and high‐throughput nature. Although CADD cannot replace clinical and molecular validation, it can, in a practical sense, assist in prioritizing variants for functional testing when an affected patient carries multiple poorly understood candidate variants, reducing waiting time for results.

However, translating this knowledge into a clinical setting is not trivial. We constructed a model based on ordinal regression of known classifications to calibrate CADD scores as a predictor of pathogenicity for gene variants in the Lynch syndrome‐associated MMR genes. Similar efforts are required to unlock the potential of CADD scores as predictors for other disorders, leading to gene‐ or disease‐specific guidelines that can help clinicians translate CADD scores into clinical practice. The threshold for “what is pathogenic” is expected to be rather different to define depending on whether the disease is caused by dominantly or recessively acting mutations, whether the disease is Mendelian or complex/multigenic in origin, and so on. Although the fact that CADD scores are largely based on conservation indicates that it may not work as well for every gene, we believe that its overall usefulness is currently unmatched by other quantitative pathogenicity estimates.

As a preliminary proof of principle, we compared the distributions of CADD scores of known pathogenic variants (from ClinVar [Landrum et al., 2013]) with the distributions of variants found in the general population (from Genome of the Netherlands [TGotN Consortium, 2014a, 2014b] and 1000 Genomes [T1GP Consortium, 2012]), for as many genes as data availability allowed. This approach can be used to estimate the predictive power of CADD scores and, thereby, provide valuable information to clinicians regarding how effective CADD scores are for predicting variant pathogenicity in the context of a specific gene. Encouragingly, out of 373 genes with sufficient data, we found 272 genes (73%) for which CADD has good predictive power (AUC of >90%).

However, this approach is currently still in development. For reliable automated calibration of CADD scores on many genes into a clinical setting, we need to consider many factors and sources of bias potentially influencing the informativity of CADD scores, such as mutation spectrum, penetrance, disorder heterogeneity, variant classification quality, classification semantics, and disorder inheritance patterns.

We conclude that in silico pathogenicity predictions are becoming powerful enough to facilitate accurate variant prioritization, at least for dominantly inherited disorders such as Lynch syndrome.

## Supporting information

Table S1. Overview of explanations according to InSiGHT why the cumulative link model based on CADD scores encountered certain false positives and false negatives
Table S2. Variants of class 2 (likely not pathogenic) for which class 5 (pathogenic) is the predicted class according to the CADD‐based model
