# Supplementary material for: Evaluation of CADD Scores in Curated Mismatch Repair Gene Variants Yields a Model for Clinical Validation and Prioritization
Source: Hum Mutat. 2015 May 20;36(7):712–9. doi: 10.1002/humu.22798 (PMC4973827; doi:10.1002/humu.22798)
Supplement: Supplementary file 1 — Table S1. Overview of explanations according to InSiGHT why the cumulative link model based on CADD scores encountered certain false positives and false negatives Table S2. Variants of class 2 (likely not pathogenic) for which class 5 (pathogenic) is the predicted class according to the CADD‐based model [file HUMU-36-712-s001.pdf]

SUPPORTING INFORMATION

Contents

1.1 CADD scaled-C scores vs. genomic coordinates 1

1.2 Primary SnpEff effect prediction vs. CADD scaled C-score 3

1.3 Overview of explanations 4

1.4 VIC justifications for class 2 where 5 was predicted 5

1.5 Comparison of CADD-based binary classifier with multifactorial likelihood . . 5

1.1 CADD scaled-C scores vs. genomic coordinates

CADD scaled-C scores vs. genomic coordinates for MMR gene variants: *MLH1*, *MSH2*, *MSH6*, and *PMS2*. The green bands are the exons. Red are InSiGHT variants, where triangles represent class 5, circles class 1, and plusses class 2-4. The black circles are variants seen in 1000 Genomes [T1GP Consortium, 2012], blue circles are seen in the Genome of the Netherlands [TGotN Consortium, 2014a; TGotN Consortium, 2014b]. The gray dots represent all potential SNVs.

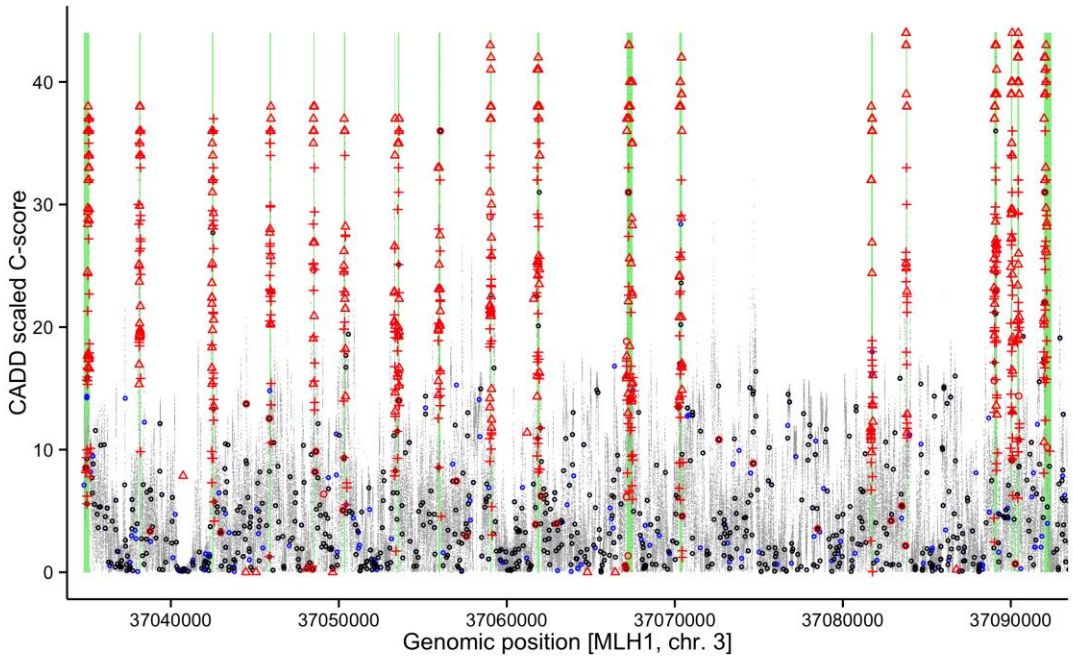

Supp. Figure S1. *MLH1*

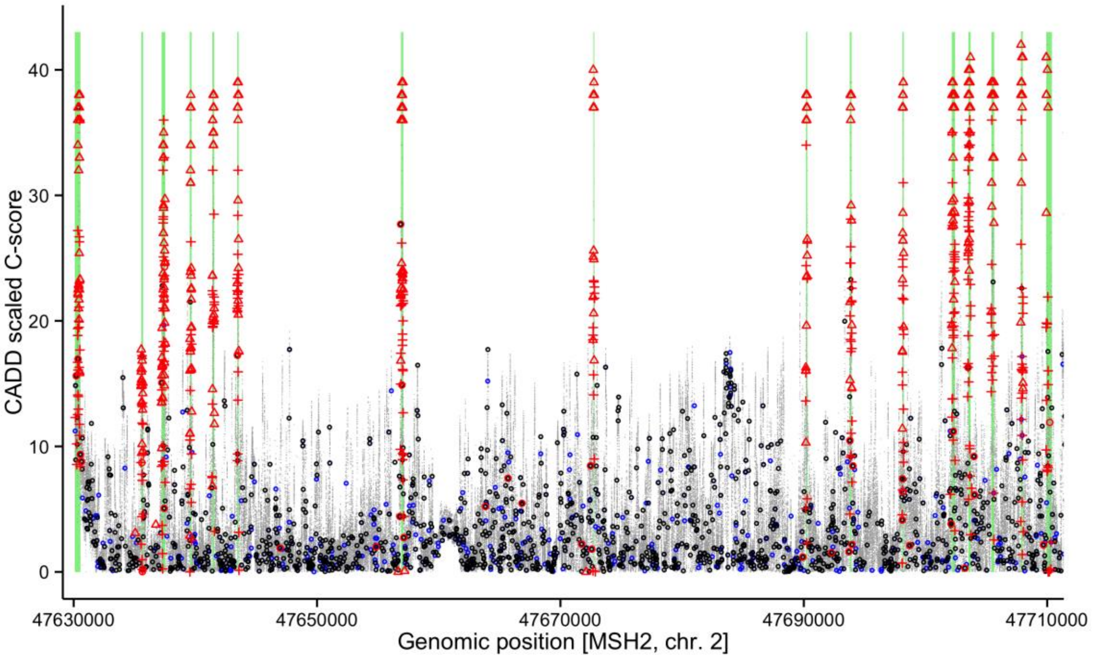

Supp. Figure S2. *MSH2*

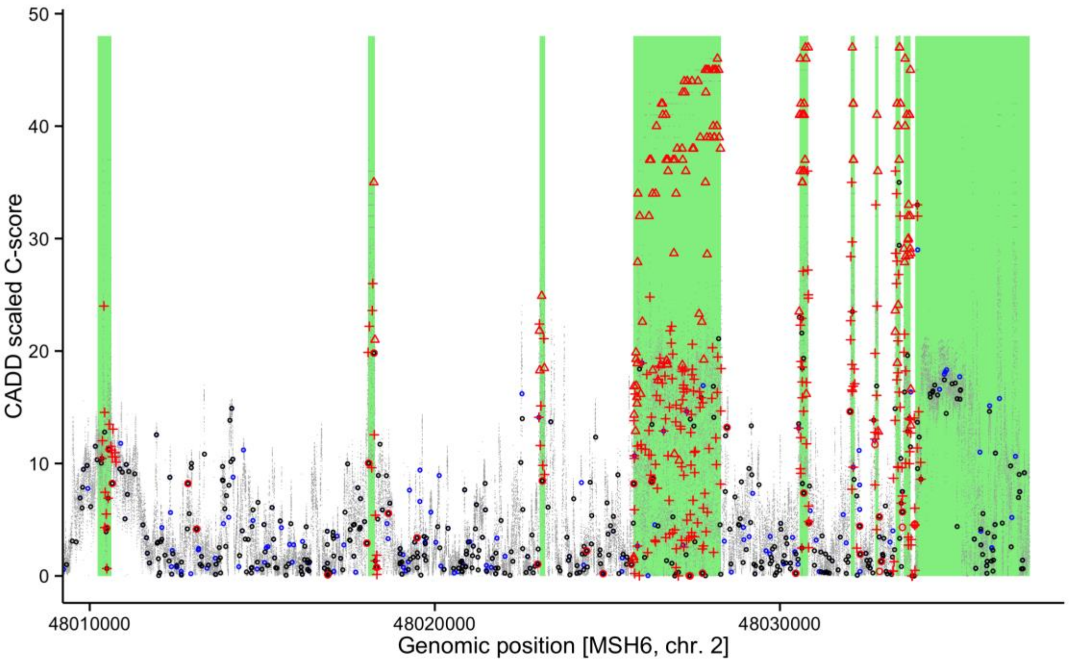

Supp. Figure S3. *MSH6*

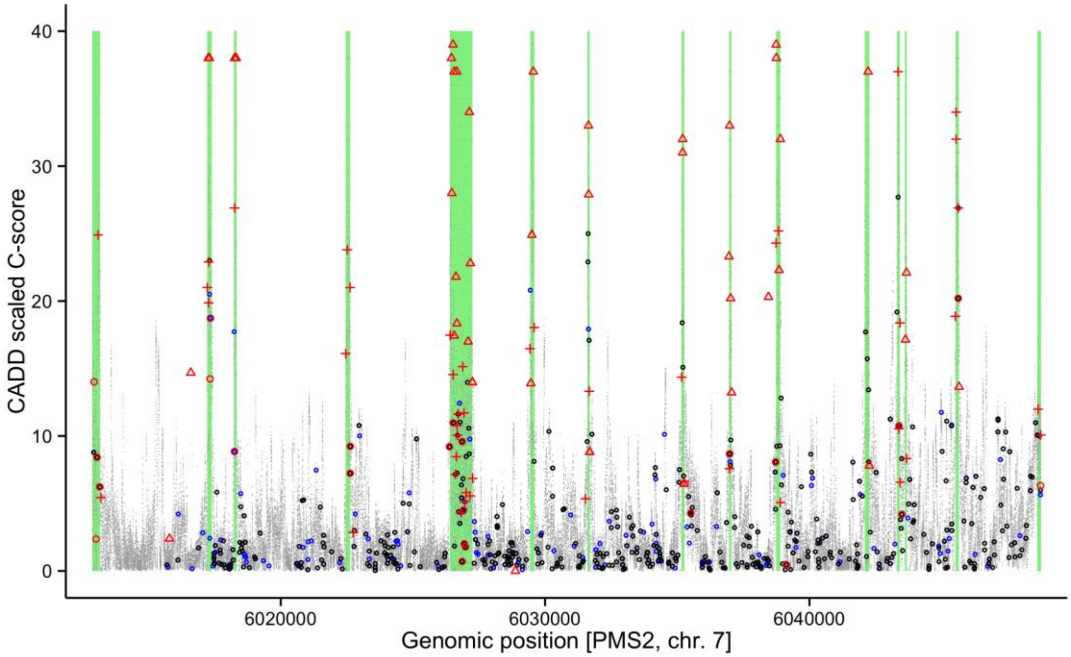

Supp. Figure S4. *PMS2*

1.2 Primary SnpEff effect prediction vs. CADD scaled C-score

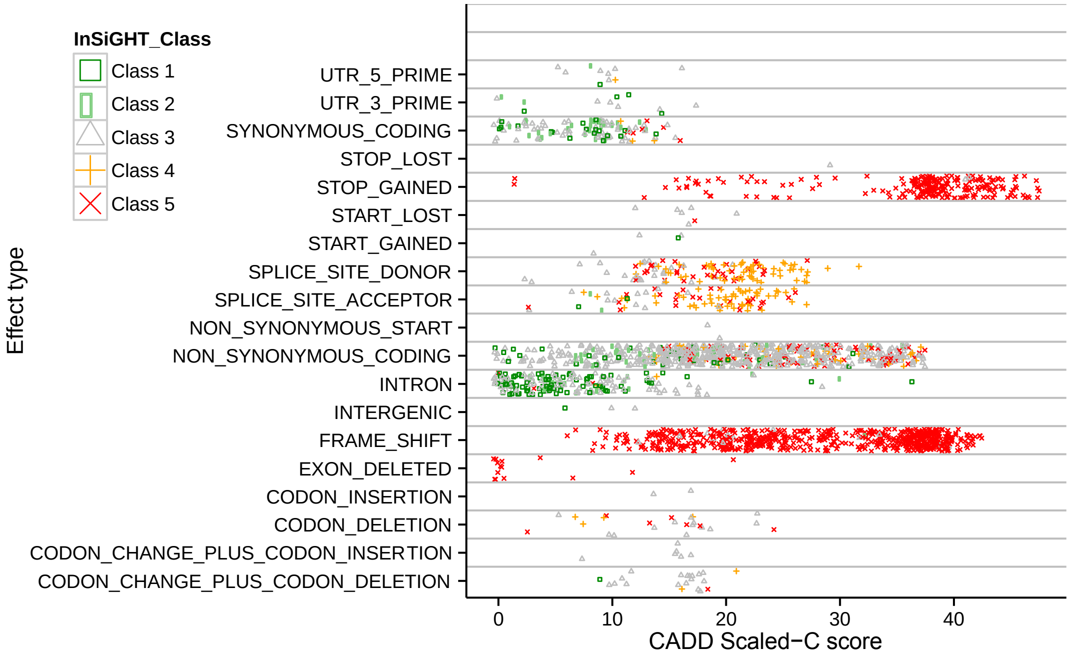

Supp. Figure S5. Primary SnpEff effect prediction vs. CADD scaled C-score, with InSiGHT classifications colored.

1.3 Overview of explanations

Supp. Table S1. Overview of explanations according to InSiGHT why the cumulative link model based on CADD scores encountered certain false positives and false negatives

Gene	Variant	InSiGHT class	CADD-based class	Explanation
MLH1	c.394G>C	1	5	Attenuated protein function, but does not cause Lynch syndrome. Multifactorial likelihood analysis posterior probability <0.001
MLH1	c.1852_1853delinsGC	1	5	Low risk, not associated with Lynch. Multifactorial likelihood analysis posterior probability <0.001
MLH1	c.803A>G	1	5	Multiple microsatellite stable tumours and does not segregate with disease. Multifactorial likelihood analysis posterior probability <0.001
MLH1	c.977T>C	1	5	Multiple microsatellite stable tumours and does not segregate with disease. Multifactorial likelihood analysis posterior probability <0.001
MLH1	c.1853A>C	1	5	Multiple microsatellite stable tumours and does not segregate with disease. Multifactorial likelihood analysis posterior probability <0.001
MLH1	c.2146G>A	1	5	Multiple microsatellite stable tumours and does not segregate with disease. Multifactorial likelihood analysis posterior probability <0.001
MLH1	c.1151T>A	1	5	Population minor allele frequency >1%
MLH1	c.2152C>T	1	5	Population minor allele frequency >1%
MSH2	c.1077-10T>C	1	5	Population minor allele frequency >1%
MLH1	c.1799A>G	1	5	Does not segregate with disease. Multifactorial likelihood analysis posterior probability <0.001
MLH1	c.790+10A>G	1	5	Does not cause splicing aberration and does not segregate with disease. Multifactorial likelihood analysis posterior probability <0.001
MSH2	c.593A>G	1	5	May be low-moderate risk, but certainly not high-risk associated with Lynch
MSH6	c.642C>A	5	1	Stop-gain variant causing protein truncation
MSH6	c.642C>G	5	1	Stop-gain variant causing protein truncation
MSH2	c.212-478T>G	5	1	Splicing aberration introduces premature termination codon (also missed by SnpEff)
MSH2	c.646-3T>G	5	1	Splicing aberration introduces premature termination codon
MSH2	c.367-480_645+644del	5	1	Deletion of Exon 3
MLH1	c.307-1420_380+624del	5	1	Deletion of Exon 4
MLH1	c.307-820_380+896del	5	1	Deletion of Exon 4
MLH1	c.381-415_453+733del	5	1	Deletion of Exon 5
MLH1	c.454-665_545+49del	5	1	Deletion of Exon 6 (raw score of 527)
MLH1	c.1039-675_1409+26del	5	1	Deletion of Exon 12 (raw score of 361)
MLH1	c.1039-2329_1409+827del	5	1	Deletion of Exon 12 (raw score of 353)
MLH1	c.1732-2243_1896+404del	5	1	Deletion of Exon 16
MSH2	c.1077-135_1276+119dup	5	1	Duplication of Exon 7 (also missed by SnpEff)
MSH2	c.1077-220_1276+6245del	5	1	Deletion of Exon 7
MSH2	c.1277-572_1386+2326del	5	1	Deletion of Exon 8 (raw score of 464)
PMS2	c.804-?_903+?del	5	1	Deletion of Exon 8
PMS2	c.804-?_2006+?del	5	1	Deletion of Exons 8-11
PMS2	c.989-296_1144+706del	5	1	Deletion of Exon 10 (raw score of 527)
PMS2	c.2276-113_2445+1596del	5	1	Deletion of Exon 14

1.4 VIC justifications for class 2 where 5 was predicted

Supp. Table S2. Variants of class 2 (likely not pathogenic) for which class 5 (pathogenic) is the predicted class according to the CADD-based model

Gene	Variant	AA change	Probability	VIC justification
MLH1	c.117-43_117-39del	*intronic*	0.99	Intronic substitution with no associated splicing aberration, tested with NMD inhibitors
MLH1	c.845C>G	A282G	0.92	Posterior probability 0.001-0.049
MLH1	c.885-24T>A	*intronic*	0.81	Intronic substitution with no effect on splicing and MAF 0.01-1%
MLH1	c.974G>A	R325Q	0.99	Posterior probability 0.001-0.049
MLH1	c.1742C>T	P581L	0.55	Posterior probability 0.001-0.049. No CMMRD phenotype with co-occurrence and MAF 0.01-1%
MLH1	c.1808C>G	P603R	0.99	Posterior probability 0.001-0.049
MLH1	c.1820T>A	L607H	0.99	Posterior probability 0.001-0.049
MSH2	c.991A>G	N331D	0.69	Posterior probability 0.001-0.049
MSH2	c.1730T>C	I577T	0.86	Posterior probability 0.001-0.049
MSH2	c.2500G>A	A834T	0.99	Posterior probability 0.001-0.049
MSH6	c.3488A>T	E1163V	0.92	MAF >1% in specific population
MSH6	c.4068_4071dup	Lys1358Aspfs*2	0.99	MAF >1% in specific ethnic group

Posterior probabilities are derived from a multifactorial likelihood analysis.

1.5 Comparison of CADD-based binary classifier with multifactorial likelihood

The multifactorial likelihood model's [Thompson et al., 2013] combination of customized MAPP + PolyPhen2 was found to perform best with an R^2 (the coefficient of determination) of 0.62 and an area under curve receiver operating characteristic (ROC-AUC) of 93%, when distinguishing classes 1 + 2 collapsed as "likely not pathogenic" versus classes 4 + 5 collapsed as "likely pathogenic". As a comparison, and not related to the cumulative link model, we performed a binary classification using CADD scores and obtained a ROC-AUC of 85%, showing that while a CADD-based binary classifier for MMR gene missense variants performs reasonably well, it does not perform as well as a disease-specific model. The data and R script for this analysis are available at http://www.molgenis.org/downloads/vdVelde_Kuiper_etal_2015/.

Supp. References

T1GP Consortium. 2012. An integrated map of genetic variation from 1,092 human genomes. Nature 491: 56–65.

TGotN Consortium. 2014a. The genome of the netherlands: Design, and project goals. European Journal of Human Genetics 22: 221–227.

TGotN Consortium. 2014b. Whole-genome sequence variation, population structure and demographic history of the dutch population. Nature Genetics.

Thompson BA, Greenblatt MS, Vallee MP, Herkert JC, Tessereau C, Young EL, Adzhubey IA, Li B, Bell R, Feng B, al. 2013. Calibration of multiple in silico tools for predicting pathogenicity of mismatch repair gene missense substitutions. Human Mutation 34: 255–265.
